# Benefits of prolonged-release pirfenidone plus standard of care treatment in patients with advanced liver fibrosis: PROMETEO study

**DOI:** 10.1007/s12072-020-10069-3

**Published:** 2020-08-19

**Authors:** Jorge Luis Poo, Aldo Torre, Juan Ramón Aguilar-Ramírez, Mauricio Cruz, Luis Mejía-Cuán, Eira Cerda, Alfredo Velázquez, Angélica Patiño, Carlos Ramírez-Castillo, Laura Cisneros, Francisco Bosques-Padilla, Larissa Hernández, Frida Gasca, Francisco Flores-Murrieta, Samuel Treviño, Graciela Tapia, Juan Armendariz-Borunda, Linda E. Muñoz-Espinosa

**Affiliations:** 1 Grupo Mexicano para el Estudio de las Enfermedades Hepáticas, Periferico Sur 4349, Local 14, Tlalpan, 14210 Mexico City, Mexico; 2 Research Unit, Tecnologico de Monterrey, Monterrey, Mexico; 3 Department of Gastroenterology, Instituto Nacional de Ciencias Médicas y Nutrición, Salvador Zubirán, Mexico City, Mexico; 4 Instituto Politécnico Nacional y Unidad de Investigación en Farmacología del Instituto Nacional de Enfermedades Respiratorias, Mexico City, Mexico; 5 Facultad de Ciencias Químicas, Benemérita Universidad Autónoma de Puebla, Mexico City, Mexico; 6 Departmento de Genética y Bioestadística, Universidad Nacional Autónoma de México, Mexico City, Mexico; 7 Instituto de Biología Molecular en Medicina y Terapia Génica, Universidad de Guadalajara, Guadalajara, Mexico; 8 Hepatology Center, Hospital Universitario, Universidad Autónoma de Nuevo León, Monterrey, Mexico

**Keywords:** Pirfenidone, Prolonged-release pirfenidone, Fibrosis, Fibrosis-regression, Fibrosis-progression, Cirrhosis, Liver, Antifibrotic, Elastography, Fibrotest

## Abstract

**Background and aims:**

Pirfenidone (PFD), an oral antifibrotic drug, has been authorized by the EMA and FDA for treatment of idiopathic pulmonary fibrosis. Few studies have addressed its use in advanced liver fibrosis (ALF). We evaluated a prolonged-release formulation (PR-PFD) plus standard of care on disease progression in ALF.

**Methods:**

281 ALF patients from 12 centers receiving PR-PFD (600 mg bid) were screened; 122 completed 1 year of treatment. Additionally, 74 patients received only standard of care regimen. Average age was 64 ± 12 years, 58% female. 43.5% had fatty liver disease (NAFLD), 22.5% viral hepatitis C (VHC), 17% autoimmune hepatitis (AIH), and 17% alcoholic liver disease (ALD). Baseline fibrosis was F4 in 74% and F3 in 26%. Antifibrotic effects were assessed by transient elastography (Fibroscan^®^) and Fibro Test^®^ (FT); Cytokines and PFD plasma levels were tracked and quality of life evaluated.

**Results:**

We found a significant reduction in fibrosis in 35% of PR-PFD patients and only in 4.1% in non PR-PFD patients. Child–Pugh score improved in 29.7%. Biochemical values remained stable; 40.6% and 43.3% decreased ALT or AST, respectively. TGFβ1 (pg/mL) levels were lower in PFD-treated patients. PFD serum concentration (µg/mL) was higher (8.2 ± 1.7) in fibrosis regression profile (FRP) patients compared to fibrosis progression profile (FPP) patients (4.7 ± 0.3 µg/mL, *p* < 0.01). 12% reported transient burning or nausea and 7% photosensitivity. Quality of life (Euro-Qol scale) improved from 62 ± 5 to 84 ± 3 (*p* < 0.001) and from 32 ± 3 to 42 ± 2 (*p* < 0.008) (FACIT scale).

**Conclusions:**

PR-PFD is efficacious and safe in ALF and associated with promising antifibrotic effects.

**Trial registration:**

Clinical trial number: NCT04099407.

## Introduction

Established advanced liver fibrosis (ALF) is considered a progressive disease, induced by several etiologies, with no definitive treatment approved by regulatory authorities. It is well established that, unless the injury is blocked or neutralized, most patients suffer complications due to liver failure and increased risk of hepatocellular carcinoma.

Evidence that liver fibrosis regresses to some extent is well-documented, both in experimental models [1, 2] and human liver disease [3, 4]. For fibrosis to regress, the underlying etiology must be treated [5]. Treatment of the underlying disease and concomitant fibrosis regression is associated with improved clinical outcomes. Fibrosis and even cirrhosis regression is characteristic of virtually all forms of liver disease. As yet, however, there is no established treatment that specifically targets liver fibrosis itself [6].

PFD is an antifibrotic drug [7] approved and with granted commercial authorization in Europe, Japan, the USA, Canada, and Mexico for the treatment of idiopathic pulmonary fibrosis (IPF). In Mexico, it has also been granted approval to treat ALF. PFD provides meaningful clinical effects on reductions in the decrease in forced vital capacity (FVC), 6-min walk distance test (6MWT), and mortality, and has improved the progression-free survival of IPF patients with mild-to-moderate disease [8]. PFD (1800–2400 mg daily) taken with food [9], and under adherence to specific access schemes [10] is well tolerated, the most common side effects being gastrointestinal discomfort and photosensitivity. PFD has a favorable benefit–risk profile and represents a suitable treatment option for patients with mild-to-moderate IPF.

This has made it an appealing candidate for other fibrotic diseases [11]. However, concerns about PFD’s potential toxicity must be addressed in diseases that have less ominous prognosis/survival rates. This is particularly pertinent for patients with liver disease, since PFD is mostly metabolized in the liver by cytochrome P450. Armendariz-Borunda et al. have shown that patients with mild ALF treated with standard-release PFD showed a reduction in inflammation and fibrosis of 30% at 12 months [12] and 67% at 24 months without significant deleterious side effects [13].

Our study rationale was to evaluate a drug with well-known effects on multiple organs and tissues, and particularly based on the knowledge that PFD inhibits TGF-β1-induced over-expression of collagen type I [11], a common pathway involved in the pathogenesis of liver cirrhosis from different etiologies.

Our study aimed to determine whether therapy with a new prolonged-release formulation of PFD (PR-PFD), specifically designed to reduce toxicity and maintain constant serum blood levels for longer duration, in combination with standard of care therapy, would result in reducing liver fibrosis and offer an even more favorable benefit–risk profile in patients with ALF.

## Patients and methods

This was a real-life, multicenter, open-label, proof-of-concept trial to determine the safety and efficacy of 12 months of treatment with PR-PFD in combination with standard of care treatment in adult patients with chronic liver disease whose fibrosis continued to progress despite abstaining from alcohol (ALD), achieving 1 year sustained virologic response (VHC), or otherwise maintaining stable disease (NAFLD, AIH) including healthy life style recommendations. Fibrosis status was determined on the basis of clinical, biochemical, ultrasound and endoscopic findings compatible with chronic damage and at least two other non-invasive methods that confirmed ALF (F3–F4).

### Inclusion and exclusion criteria

**Fig. 1:**
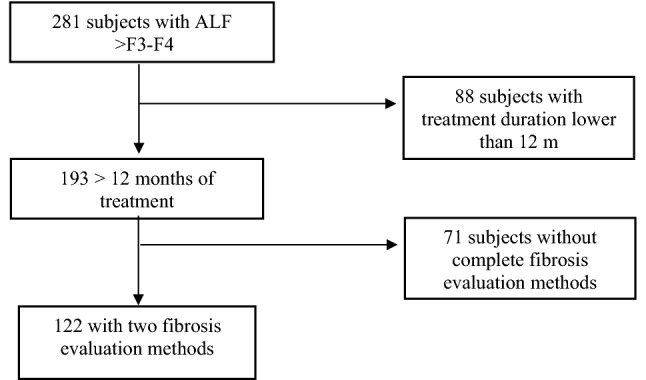
Inclusion and exclusion criteria

Two hundred and eighty-one patients treated with PR-PFD were identified in 12 medical centers. Patients were excluded if they used hepatotoxic drug, decompensation in the previous 6 months, prior history of malignancy, active infectious processes not of a self-limited nature, hemolysis, alpha-fetoprotein > 100 ng/L, pregnancy, and alcohol or intravenous drug abuse within the previous year, were on active treatment with PR-PFD less than 12 months (*n* = 88) or lacked baseline and final fibrosis measurements (*n* = 71) (Fig. 1).

The study group comprised the remaining 122 patients. 71 were female and 51 male. Etiology was ALD in 21 patients (17%), AIH in 21 (17%), HCV in 27 patients (22%), and NAFLD in 53 (43%). All HCV patients received previous treatment for virologic disease and still had advanced fibrosis despite sustained viral response (SVR) at 12 months. All patients with AIH were receiving the lowest possible dose of prednisone and/or azathioprine in order to induce low necroinflammatory activity or biochemical remission. Considering the advance fibrosis stage (F3–F4) of our patient’s cohort, none of them presented ALT or AST values higher than twofold ULN.

Patients were 52–76 years of age (mean 64 ± 12 years old). All subjects had a complete medical history, lab results, hepatic ultrasound, and upper-gastrointestinal endoscopy at baseline and 12 months (M12) after pharmacological intervention. Patients were negative for hepatocellular carcinoma (HCC) as determined by hepatic ultrasound and had the following lab values: hematocrit > 30%, hemoglobin > 10 g/dL, platelet count > 30 × 10^9^/L, white blood cell count > 3 × 10^9^/L, and serum creatinine level < 1.5 mg/dL. Normal TSH or hypothyroidism under hormone replacement therapy was also required for inclusion. Concomitant disease was detected in 53 patients (43%) including diabetes mellitus (*n* = 16), systemic hypertension (*n* = 7), dyslipidemia (*n* = 21), osteoarthrosis (*n* = 11), and inflammatory bowel disease (*n* = 1). None of them presented decompensation or required additional treatment during the whole duration of the study.

Ninety were at stage F4 and 32 at F3. Seventy-seven (85.6%) of 90 cirrhotic subjects were compensated and 13 decompensated (12 with ascites and 5 with previous esophageal variceal bleeding). All decompensated patients were stable with diuretics. Those with previous variceal hemorrhage received propranolol to achieve a ≥ 20% reduction in baseline heart rate and completed a variceal band ligation program. Patients with large varices without previous hemorrhage also received betablockers and/or variceal band ligation as primary prophylaxis. None had overt portal systemic encephalopathy. 65 had grade A Child–Pugh scores (72.2%), 23 had grade B (25.6%), and 2 had grade C (2.2%). 27 had a MELD score lower than 9 (30%), and 63 had a MELD of 10–19 (70%). 43 patients (47.7%) were at stage 1 cirrhosis (without esophageal varices), 30 (33.3%) at stage 2 (esophageal varices without previous bleeding); 12 (13.3%) at stage 3 (with clinical- and ultrasound-detected ascites) and 5 (5.7%) stage 4 (previous esophageal variceal bleeding). None suffered gastric variceal bleeding during the study.

For the purpose of evaluation of fibrosis progression evolution, we also included a control group of patients with advanced liver fibrosis (*n* = 74) with standard of care treatment. Baseline demographic and clinical findings compared to patients receiving standard of care plus PF-PFD are depicted in Table 1.

**Table 1:** Patient baseline demographic and clinical characteristics

Characteristics	Control group (*n* = 74)	Pirfenidone final group (*n* = 122)	Pirfenidone pre-selection group (*n* = 281)
Age (years)	59 ± 9	64 ± 12	62 ± 12
Female [*n* (%)]	54 (73%)	71 (58%)	138 (49%)
Cirrhosis cause			
HCV [*n* (%)]	30 (40.5%)	27 (22%)	71 (25.3%)
HCB [*n* (%)]	0	0	5 (1.8%)
NAFLD [*n* (%)]	27 (36.5%)	53 (43%)	113 (40.2%)
ALD [*n* (%)]	8 (11%)	21 (17%)	45 (16.1%)
Autoimmune [*n* (%)]	9 (12%)	21 (17%)	47 (16.7%)
Fibrosis METAVIR score			
F3 [*n* (%)]	3 (4%)	32 (26%)	62 (22%)
F4 [*n* (%)]	71 (96%)	90 (74%)	219 (78%)
Child–Pugh score			
A [*n* (%)]	68 (96%)	65 (72.2%)	208 (74%)
B [*n* (%)]	3 (4%)	23 (25.6%)	65 (23%)
C [*n* (%)]	0	2(2.2%)	8 (3%)
Cirrhosis stage			
1 [*n* (%)]	39 (54.9%)	43 (47.7%)	57 (26.4%)
2 [*n* (%)]	29 (40.8%)	30 (33.3%)	110 (50.9%)
3 [*n* (%)]	3 (4.3%)	12 (13.3%)	35 (16.2%)
4 [*n* (%)]	0	5 (5.7%)	14 (6.5%)
MELD score			
≤ 9	45 (63%)	27 (30%)	130 (46%)
10–19	24 (34%)	63 (70%)	143 (51%)
≥ a 20	2 (3%)	0	8 (3%)

### Study design and treatment regimens

Treatment consisted of 600 mg tablets of PR-PFD (Kitoscell LP^®^ in Mexico and authorized by the local Drugs Agency (COFEPRIS) of the Ministry of Health). Patients were instructed to take medication orally, every 12 h, after breakfast and dinner. All participants were required to adhere to a standard of care that included nutritional support, quarterly medical evaluation to review lab results and adjust medications, bi-annual liver ultrasound, and annual upper-gastrointestinal endoscopy.

### Clinical and laboratory evaluation

Blood counts and liver function tests (bilirubin, albumin, prothrombin time expressed as INR, serum transaminases, glucose, and creatinine) were measured at 12-week intervals. Patient’s somatometric measurements (height and body weight), vital signs, and frequency of adverse events (AEs) were recorded. Liver enzymes were scored as stable, improving, or worsening. FT results were analyzed at a central laboratory.

### Study end points

The primary efficacy endpoint was a reduction of fibrosis score by at least 30% either in FT units or kilo Pascals (kPa) according to hepatic elastography or a reduction of 1 point on the METAVIR scale. Secondary efficacy endpoints included improvement in ALT and/or AST, albumin, serum concentrations of TGFbeta, IL-1 and IL-6 and endothelin, and Child–Pugh and MELD scores. Worsening MELD was defined as switching from a lower-score to a higher-score and improving as switching from a higher-score to a lower-score, where Group 1 was MELD ≤ 9, Group 2 10–19, and Group 3 > 20.

Primary safety endpoints included clinical side effects, blood profile abnormalities, overall survival, and PK findings. Secondary safety endpoints included quality-of-life scores.

### Evaluation and classification of fibrosis outcomes

Fibrosis regression profile (FRP): decreases greater than 30% in FT score or 30% in kPa in liver stiffness measurement (LSM) or decreasing 1 point on the METAVIR score comparing baseline and M12 measurements.

Fibrosis-stabilization profile (FSP): stable FT results or kPa measurements (variations lower than 30%) or METAVIR score.

Fibrosis progression profile (FPP): increases greater than 30% in FT score or kPa or increasing 1 point on METAVIR.

### Specific evaluation and classification of biochemical outcomes

#### Biochemical markers

Blood parameters determined after overnight fasting included: albumin, prothrombin time, total bilirubin, ALT, AST, AP, and GGT, measured in fresh serum within 8 h of collection on an automated biochemistry analyzer (Hitachi 917; Roche Diagnostics). α2 macroglobulin, apolipoprotein-A1, and haptoglobin levels were assayed by nephelometry (Image; Beckman Coulter).

#### Fibro test^®^

FT measurements were done on fresh serum, blinded to the clinical data and according to the recommended pre-analytic and analytic methods. The laboratory followed the pre-analytical and analytical recommendations required to obtain the fibrosis marker score FT [14]. FT provides a quantitative estimate of liver fibrosis ranging from 0.00 to 1.00. The semi-quantitative analysis used predetermined cut-offs equivalent to the standard cut-offs for non-cirrhotic METAVIR stages; for FT: F0 (0–0.28), F1 (> 0.28–0.48), F2 (> 0.48–0.58) and F3 (> 0.58–0.74). Cirrhotic stages were as follows: F4.1 (> 0.74–0.85), F4.2 (> 0.85–0.95) and F4.3 (> 0.95–1.00). A significant decrease/increase in fibrosis was defined as a decrease/increase of 30%. This test was not performed on control group patients.

#### Hepatic elastography

Transient elastography (TE) was performed according to published recommendations [15] using the Fibro-Scan^**®**^ M probe. LSM was expressed in kPa. Only procedures with at least 10 validated measurements, a > 60% success rate, and an interquartile range < 30% of the median were considered reliable. The semi-quantitative analysis used predetermined cut-offs equivalent to the standard cut-offs for non-cirrhotic METAVIR stages. F0 (0–5 kPa), F1 (> 5–7.1 kPa), F2 (> 7.1–9.5 kPa), and F3 (> 9.5–12.5 kPa). Cirrhotic stages were as follows: for F4.1 (> 12.5–20 kPa), for F4.2 (> 20–50 kPa), and for F4.3 (> 50–75 kPa). A significant decrease/increase in fibrosis was defined as a decrease/increase of 30% in kPa units. Patients with ascites were offered real-time shear wave elastography (Aixplorer, Supersonic Imagine), a more reliable evaluation method for these patients [16].

#### Cytokines and pirfenidone

Serum concentrations of interleukin 6 (IL-6), transforming growth factor beta (TGF-β1), endothelin 1 (ET-1), and tumor necrosis factor alpha (TNF-α) were quantified by ELISA in an automated EIA analyzer Coda Microplate System (Bio-Rad Laboratories, Inc., Hercules, California, USA) [17] and values normalized against serum of healthy volunteers (*N* = 32) with normal liver function and LSM < 5 kPa (F0) and non-treated cirrhotic patients (*n* = 31) with LSM > 12.5 kPa (F4). A subgroup of 65 PR-PFD treated patients was included for cytokines measurements. PFD plasma levels were measured in fasting conditions and 2 h after standard breakfast (around 400 kcal) and medication intake, using an HPLC with UV detection method.

### Evaluation of safety profile

Monitoring for safety and toxicity was performed throughout the study. When necessary, appropriate medical intervention was provided. PR-PFD was suspended in any patient who experienced severe clinical (e.g., photosensitivity) or laboratory toxicity (grade 3, modified Aids Clinical Trials Group graded toxicity scale), until toxicity returned to baseline. PR-PFD was permanently discontinued if toxicity persisted or if a patient experienced life-threatening (WHO grade modified ACTG graded toxicity scale) toxicities.

### Quality-of-life assessment

All patients filled out the Euro-Qol Index survey, including the visual analog scale evaluation at baseline and at 12 m. We also incorporated the nonutility-based Short Form-36v2 survey, which provides a detailed profile of health-related quality of life [18].

### Statistical data analysis

Statistical evaluation was performed using IBM SPSS^®^ version 22. Differences in laboratory parameters such as ALT, AST or cytokines were compared between groups using Student’s *t* test or Mann–Whitney *U* test or one-way ANOVA test when applicable. Given that the data of cytokines and PFD showed a non-normal distribution, they were analyzed by a generalized linear model (GzLM) with maximum likelihood as method of estimation. The marginal means were estimated for each treatment and compared by pairs of groups by Bonferroni test. Significant statistical differences were considered at *p* < 0.05. Sample size calculation was based on previous data from our local experience and the following assumptions: (a) mean expected baseline elastography score of 27.4 kPa (b) standard deviation of 15.7; (c) expected estimated-fibrosis reduction rate higher than 30% (delta); (d) alpha error of 1%; (e) an accepted beta error of 10% (power = 90%); (f) two tails. The final number of patients required to find a significant difference using the G power statistical program [19] was 61 patients.

## Results

A flowchart of the total study population is shown in Fig. 1. Only 122 participants with ALF (F3-F4) completed study-medication for at least 12 months. As expected in an open, real-life trial, some patients (*n* = 71) did not provide the fibrosis evaluation methods. However, an intention-to-treat (ITT) analysis showed no statistically significant differences between patients included and those who were not included (baseline data in Table 1).

### Fibrosis regression

**Fig. 2:**
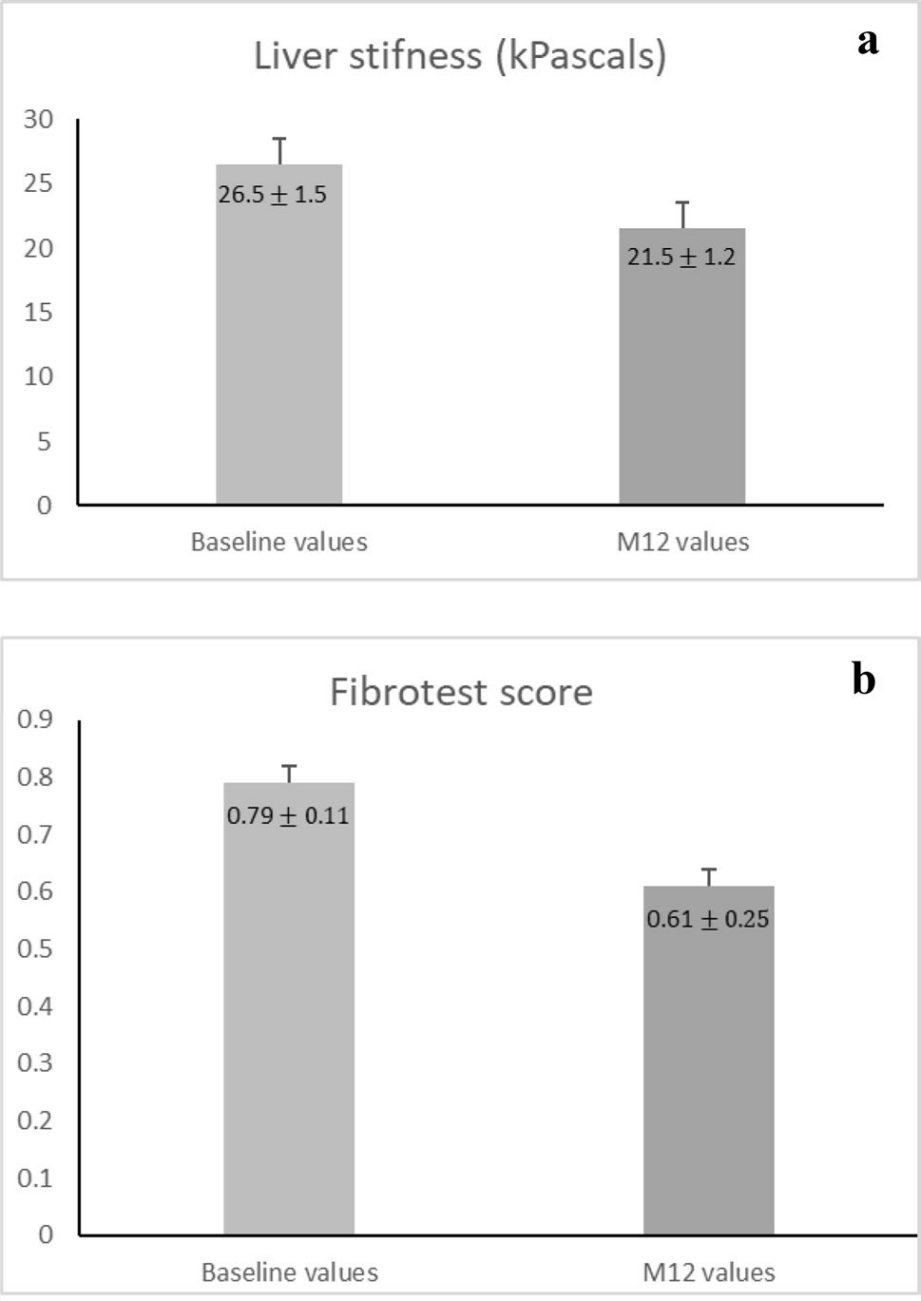
**a** Statistical significance was analyzed by Student’s *t* test, *p* = 0.023, compared to baseline values. **b** Statistical significance was analyzed by Student’s *t* test, *p* = 0.0001, compared to baseline values

A significant reduction in LSM, 26.5 ± 2.0 kPa versus 21.5 ± 2.0, *p* < 0.05 and FT score, 0.79 ± 0.01 versus 0.61 ± 0.02, *p* < 0.0001 was observed in the total population (Fig. 2). In the PR-PFD group, 35.2% (*n* = 43) presented a FRP; 46.7% (57 patients) a FSP; and 18.0% (22 patients) a FPP. In the control group, only 4.1% (*n* = 3) presented a FRP; 78.3% (58 patients) a FSP; and 17.6.0% (13 patients) a FPP (Table 2). LSM score increased from 21.6 ± 0.0.98 kPa to 24.9 ± 1.07.

### Clinical outcomes

One hundred and seven patients finished at Grade A, 15 at Grade B, and 0 at Grade C; 8 improved from Grade B to Grade A and 2 from Grade C to Grade B/Grade A (Table 2). 36 (29.5%) decreased or improved (*p* < 0.001) and 13 (10.7%) increased or worsened by 1 point, while 73 (59.8%) remained stable.

**Table 2:** Liver fibrosis evolution according to elastography

	Control group (*n* = 74)	Pirfenidone group (*n* = 122)
According to liver stiffness by hepatic elastography measurement		
Fibrosis progression profile (FPP) [*n* (%)]	13 (17.6%)	22 (18.3%)
Fibrosis-stable profile (FSP) [*n* (%)]	58 (78.3%)	57 (46.7%)
Fibrosis regression profile (FRP) [*n* (%)]	3 (4.1%)	43 (35.2%)

A significant decrease/increase in fibrosis was defined as 30% of change, compared to baseline values

A higher number ended in MELD score group 1 (≤ 9), and only 2 (1.6%) progressed to > 20 (Tables 3 and 4); 88 (72.1%) remained stable, 16 (13.1%) worsened, and 18 (14.8%) improved MELD score.

**Table 3:** Child–Pugh class and MELD evolution in F4 patients

	Control group (*n* = 71)		Pirfenidone group (*n* = 90)	
	Baseline	Final	Baseline	Final
Child–Pugh A	68 (97%)	69 (97%)	65 (72.2%)	75 (83.3%)
Child–Pugh B	3 (4)	2 (3%)	23 (25.5%)	15 (16.7%)
Child–Pugh C	0 (0%)	0 (0%)	2 (2.2%)	0 (0.0%)
Child–Pugh Score	5.3 ± 0.6	5.2 ± 0.5	5.8 ± 1.2	5.7 ± 0.9
MELD risk categories				
Score ≤ 9	45 (63%)	38 (54%)	27 (30%)	33 (36.7%)
Score 10–19	24 (34%)	32 (45%)	63 (70%)	55 (61.1%)
Score > 20	2 (3%)	1 (1%)	0 (0.0%)	2 (2.2%)
Average	8.8 ± 3.0	9.3 ± 2.6	10.2 ± 3.1	10.0 ± 3.1

**Table 4:** Child–Pugh score and MELD sub-analysis in PR-PFD group

Child–Pugh	Population	Percentage (%)	MELD	Population	Percentage (%)
Stable score	73	59.8	Stable	88	72.1
Increased score	13	10.7	Worsening	16	13.1
Decreased score	36	29.5	Improving	18	14.8

Significant changes were defined as 1-point increase or 1-point reduction in Child–Pugh score

Worsening was defined as switching from group 1 (score of 6–9) to group 2 (score 10–19) or group 3 (score > 20) and improving as reducing allocation to a lower-score group

Blood tests evolution remained stable in 75 patients (61.5%). Only nine patients changed Cirrhosis stages, 7 from Stage 3 to Stage 2 (*p* = 0.03) and 2 from Stage 3 to Stage 4. (One presented active hematemesis; one had melena with active variceal hemorrhage confirmed by endoscopy.) None had multiple liver-related AE and/or other organ involvement. None suffered gastric variceal bleeding. None presented uncontrolled encephalopathy or uncontrolled ascites. Noteworthy, ALT and AST serum levels remained stable in 53 (43.4%) and 56 (45.9%), respectively; 49 (40.2%) and 53 (43.4%) decreased transaminase serum levels (*p* < 0.00005) and only 20 (16.4%) and 13 (10.7%) increased.

Body weight and BMI values decreased at month 12, whereas global biochemical data remained stable (Table 5).

**Table 5:** Somatometric and biochemical evolution

Characteristics	Control group (*n* = 74)		Pirfenidone group (*n* = 122)	
	Baseline	M-12	Baseline	M12
Body weight (kg)	70.1 ± 7.3	71.2 ± 6.96	78.7 ± 17.9	71.5 ± 11.3
BMI (kg/m^2^)	27.98 ± 7,19	28.41 ± 6.96	31.2 ± 7.6	28.9 ± 3.6
Hemoglobin (g/dL)	13.6 ± 1.4	13.7 ± 1.2	13.9 ± 2.2	13.6 ± 1.6
Leukocytes (× 10^3^)	5.2 ± 1.5	4.8 ± 1.1	4.5 ± 1.9	4.5 ± 1.8
Platelets (× 10^3^)	128 ± 5	131 ± 6	103 ± 8	101 ± 8
Bilirubin (mg/dL)	1.1 ± 0.5	1.1 ± 0.5	1.6 ± 1.0	1.3 ± 0.5
Albumin (g/dL)	3.8 ± 0.4	3.9 ± 0.4	3.6 ± 0.6	3.5 ± 0.5
P.T. (INR)	1.2 ± 0.2	1.2 ± 0.2	1.3 ± 0.3	1.3 ± 0.2
ALT (mg/dL)	63 ± 4	66 ± 2	47 ± 5.0	45 ± 4.2
AST (mg/dL)	73 ± 4	78 ± 2	58 ± 5.9	54 ± 4.0
Alkaline phosphatase (mg/dL)	96 ± 4	95 ± 4	194 ± 17.9	187 ± 19.3
GGT (mg/dL)	69 ± 8	66 ± 5	124 ± 15.7	148 ± 16.9

Values are mean ± SD

*NA* not available data

#### Endoscopic findings

Absence of esophageal varices improved from 26 patients (21.3%) to 38 (34.9%) at M12. That is, esophageal varices disappeared in 12 patients. At baseline, 62 (50.8%) had small and 34 (27.9%) had large varices. In the 109 who accepted endoscopy at M12, 25% had small varices and 40% had large varices. Although varices increased in size in 10, only 2 had variceal hemorrhage.

#### Liver ultrasound major findings

Chronic liver disease was confirmed at baseline in 122 patients, 53 patients had splenomegaly and 12 patients had mild ascites. At M12, 68 had splenomegaly and 6 had ascites.

#### Cytokines serum concentration

Cytokines levels in the serum of healthy subjects were significantly lower compared to those in PR-PFD-non-treated cirrhotics and our study group (Table 6). TGF-β1 concentrations were significantly higher in cirrhotic patients without treatment (99.6 ± 7.5 pg/mL) compared to control subjects (46.2 ± 2.5 pg/mL, *p* < 0.01) and patients under active treatment with PR-PFD (61.8 ± 2.3 pg/mL, *p* < 0.05). Interleukin-6 (10.5 ± 1.04 versus 13.9 ± 1.7, pg/mL, *p* < 0.05) and endothelin-1 (17.8 ± 1.6 versus 22.1 ± 3.41, pg/mL, *p* < 0.05) were also significantly decreased in PR-PFD-treated patients compared to non-treated cirrhotics (Table 6).

**Table 6:** TGF-β1 serum concentrations

Parameters	Healthy subjects (*n* = 32)	Subjects with cirrhosis without treatment (*n* = 31)	Subjects with fibrosis and treatment (*n* = 65)
TGF-β1 (30–60 pg/mL)	46.2 ± 2.5	99.6 ± 7.5^a^	61.8 ± 2.3^a,b^
IL-6 (30–60 pg/mL)*	3.9 ± 0.7	13.9 ± 1.7^a^	10.5 ± 1.04^a,b^
TNF-α (1.2–15.3 pg/mL)	8.7 ± 1.5	25.97 ± 2.3^a^	23.0 ± 1.06^a^
Endothelin-1 (1.2–15.3 pg/mL)	13.8 ± 2.2	22.1 ± 3.4^a^	17.8 ± 1.6^a,b^

Statistical significance was analyzed by a generalized linear model with maximum likelihood as method of estimation and Bonferroni test

^a^*p* < 0.01 compared to healthy subjects

^b^*p* < 0.05 compared to cirrhotic patients, non-treated with pirfenidone

#### Pharmacokinetic data

PFD plasma concentrations measured at 12 months and under continuous treatment were 6.7 ± 0.78 µg/mL pre-prandial and 8.9 ± 0.74 µg/mL post-prandial indicating stable medication levels. Notably, FRP patients had higher fasting PDF plasma levels (8.2 ± 1.7 µg/L) than FSP (6.2 ± 1.4 µg/L, *p* < 0.05) or FPP patients (4.7 ± 0.3 µg/L, *p* < 0.001). Delta values between fasting and 2-h post-prandial were lower in FRP patients (Table 7).

**Table 7:** Pirfenidone serum concentrations

Pirfenidone serum levels	Fibrosis progression profile (FPP)	Fibrosis-stable profile (FSP)	Fibrosis regression profile (FRP)
Fasting sample (µg/mL)	4.7 ± 0.3	6.2 ± 1.4	8.2 ± 1.7^a,b^
2-h post-prandial sample (µg/mL)	8.2 ± 0.1	9.0 ± 1.3	9.3 ± 1.4

Statistical significance was analyzed by a generalized linear model with maximum likelihood as method of estimation and Bonferroni test

^a^*p* < 0.01 compared to fibrosis progression profile subjects

^b^*p* < 0.05 compared to fibrosis-stable profile subjects

#### Safety profile

The side effects seen in our study cohort are described in Table 8.

**Table 8:** Side effects list

Characteristics	Pirfenidone final group (*n* = 122)	Pirfenidone pre-selection group (*n* = 281)
Nausea	12 (9.8%)	37 (13.2%)
Dyspepsia	10 (8.2%)	30 (10.7%)
Diarrhea	4 (3.3%)	12 (4.2%)
Rash	9 (7.4%)	17 (6.0%)
Death	0 (0%)	8 (2.8%)

*NA* not available data

#### Survival curve

An intention-to-treat analysis of the total population taking PF-PFD was carried out even if they did not complete the 12-month treatment period or the whole evaluation (281 patients). There were 8 deaths: 4 were not liver related; 1 followed massive esophageal hemorrhage; 3 were due to progressive liver failure. There were 0 deaths in the study group. 

#### Quality of life

According to 75 self-reported questionnaires, quality-of-life scale improved from 62 ± 5 to 84 ± 3 (*p* < 0.001) and FACIT scale from 32 ± 3 to 42 ± 2 (*p* < 0.008).

## Discussion

This first-ever, real-life, multicenter, open-label study shows a significant effect of PR-PFD in reducing ALF. Significant beneficial effects were also noted in several clinical, biochemical, and molecular parameters.

Treatment with a prolonged-release formulation of PR-PFD, in combination with a standard of care regimen, resulted in either a FRP or a FSP in the vast majority (35% and 46.7%, respectively), confirming PR-PFD’s significant antifibrotic effects in ALF and extending previous findings [24, 25]. A remarkable difference was observed when compared to only 4.1% FRP seen in the control group, receiving the standard of care regimen.

While some clinicians used to consider cirrhosis irreversible, there are well-documented cases of reversibility of ALF [3], largely correlated with effective treatment of the particular etiology [4, 5]. However, in some patients, fibrosis progresses despite effective treatment. Accordingly, there is a need for ongoing research for treatments directed at fibrosis itself.

This has stimulated evaluation of new antifibrotic drugs [6] in hepatic disorders. Promising studies, like those of Armendariz-Borunda et al. [12, 13] show reduction in both inflammation and fibrosis with negligible side effects. Nonetheless, more rigorous studies concerning PFD’s safety are mandatory, particularly in ALF and given that drug toxicity could increase due to liver impairment.

In our study, PR-PFD was safe and generally well tolerated. Treatment adherence was high, partially explained by patient motivation and a friendly dosing schedule. The type and frequency of AE were consistent with the known safety profile of PFD [8, 10, 11], AEs were typically mild or moderate, and none led to permanent treatment discontinuation. No deaths were detected in the 12-month treatment population and few overall deaths in the whole cohort of eligible population, only half of those liver related.

Our data on blood plasma levels allowed us to evaluate adherence to treatment and better understand the relationship between plasma levels and fibrosis response. In addition to confirming detectable plasma levels in all participants, we identified higher and significant PFD-plasma levels in FRP patients than in FPP patients, suggesting better drug impregnation in this subgroup of responders. Interestingly, delta values between fasting and 2-h post-prandial levels were similar in FRP patients but different in FPP patients, indicating a possible metabolic effect at the hepatic cytochrome level. In addition, this is the first study that provides information of medication levels in a cirrhotic population.

Because the presence of food significantly reduces the extent of absorption of PFD [20], all participants were instructed to take medication after food to reduce known side effects. The plasma levels we found do not point to an inhibitory effect of absorption in FFP patients, but rather a different hepatic metabolism. Around 60% of PFD is bound to plasma proteins, especially to albumin [11], which was found in similar levels in FPP and FRP patients. Since up to 50% of the drug is metabolized by the hepatic CYP1A2 enzyme system to yield 5-carboxy pirfenidone, the inactive metabolite, it is possible that FRP patients have a different enzymatic activity. It is important to keep in mind that our patients were not using fluvoxamine, amiodarone, or propafenone, considered inhibitors of the enzymatic activity of CYP1A2. Similarly, our cohort was not using ciprofloxacin, another CYP1A2 inhibitor. Moderate inducers of CYP1A2, such as tobacco smoking or omeprazole, can reduce the circulating plasma levels of the drug [21]. While none of our patients smoked tobacco, some were taking PPIs. Nevertheless, there was no difference between the FPP and FRP subgroups.

Armendariz-Borunda et al. [22], have suggested that the effectiveness of PFD could be influenced by inherited genetic polymorphisms that increase the risk of developing ALF in some patients. The different PK plasma levels in our study confirm that possibility. Similar clinical trials must consider PFD plasma levels to improve our understanding.

It is assumed that cytokines play central roles in the progression from chronic liver injury to fibrosis/cirrhosis and several pro-inflammatory cytokines (such as IL6, and TNFα) correlate with disease severity [23]. In addition, evidence suggests that hepatic stellate cells express the highest levels of endothelin receptors that may contribute to hepatic sinusoidal tone and therefore portal hypertension [24]. In our study, TGFβ1 serum levels were significantly decreased in PR-PFD-treated patients compared to untreated patients. We also found significant reductions in IL6 and endothelin levels. This suggests that cytokine changes may help predict liver fibrosis evolution.

One of the remarkable facts of our study was the inclusion of patients with different ALF etiologies, as it happens in real life. Our population was recruited from both academic hospitals and ambulatory care units in order to mimic the general population profile, which could increase the external validity of our findings. We understand that fibrosis progression may behave differently in relation to etiology of the liver disease, particularly in patients with AIH. However, according to Hartl et al. [25], transient elastography has been shown to be a reliable tool to monitor disease course in AHI.

In relation to higher relative frequency of HCV among controls (40.5%) compared to PFD-treated population (22%), described in Table 1, we consider this finding is explained because our selection criteria required patients with HCV-treatment and at least 1 year of sustained viral response.

A potential limitation is the absence of liver biopsy, a method with many drawbacks, such as sampling error, cost, and risk of complications [26]. This is mitigated, first, because that, too, mimics the population typical of ambulatory care where non-invasive methods of estimating liver fibrosis are frequently used [27]. Second, non-invasive assessments have been developed and adopted in international management guidelines [15]. LSM by different elastographic methods or magnetic resonance has been found to have an excellent correlation with biopsy findings and is increasingly used in clinical trials. Similarly, FT has been extensively evaluated in patients with chronic liver disease due to diverse etiologies and also has a high correlation biopsy findings, particularly in patients with ALF. According to Lau-Corona et al. [28], FT could enhance our ability to assess differences in fibrosis scores in clinical studies and improve our understanding of fibrosis progression. Recently, Mauro et al. [29], evaluated the value of portal pressure, liver stiffness, and enhanced liver fibrosis score measurements to predict fibrosis regression according to paired liver biopsies before and after sustained viral response (SVR) in recurrent HCV patients. They concluded that the dynamic changes in LSM accurately predict the presence of ALF and clinically significant portal pressure 1 year after SVR and thus can be used as reliable monitoring strategies. Chalasani et al. [30], confirm the utility of non-invasive markers in ALF patients by showing that longitudinal changes in non-invasive measures of fibrosis correlate with improvements in histologic fibrosis and therefore can serve as surrogate end points in clinical trials. Third, we used two validated, non-invasive methods.

It is worth mentioning that we observed an 11% reduction in body weight throughout the duration of the study in the pirfenidone-treated group. Since diet and exercise are known factors to influence disease progression/regression, and even portal pressure reduction, we consider that controlling these factors should be part of the commitments of the standard of care.

Another important finding is significant improvements in liver function and liver enzymes. Child–Pugh is considered a strong bedside prognostic indicator in advanced liver disease, often used with MELD to determine need and priority for transplant [18]. Here, the vast majority improved both scores or remained steady, which correlates with an increase in serum albumin, another clinical prognostic indicator.

Contrary to what might be expected regarding possible liver toxicity, no patients’ AST/ALT levels increased X5 upper limit of normal (ULN). Rather, we identified a significant decrease in 40.6% and 43.3% in ALT and AST serum levels, demonstrating a favorable safety profile. In Angulo et al. study [31], which reports a high proportion of AE in 24 primary sclerosing cholangitis patients, treatment was with standard-release PFD and at a much higher daily dose (2400–3600 mg). Key strategies to prevent and manage common PFD AEs have been described to maximize adherence and prevent AEs [10].

Regarding the suitability of the PFD formulation that we used in our study, PR-PFD offers a lower *C*_max_ and longer *T*_max_ and half-life pharmacokinetic profile compared to standard-release PFD [20].

PR-PFD association with lower PFD *C*_max_ values (Poo JL, et al. Pharmacokinetics of the antifibrotic drug pirfenidone in Child–Pugh A and B cirrhotic patients compared to healthy age-matched controls. Journal of Hepatology 2016;64:S213–S424) may explain the excellent tolerability in our study. Also promising is that the self-assessment questionnaires indicated significant QOL effects, mainly described as improvement in energy and reduction in fatigue.

In conclusion, prolonged-release PFD administration in conjunction with standard of care treatment in patients with advanced liver fibrosis provides beneficial effects that warrant additional studies. Our data suggest that inflammation and liver stiffness could be ameliorated by PFD-treatment, a finding needing confirmation in a placebo-controlled clinical trial.
